# Human Sexual Development in the Somatic and Psychosexual Context

**DOI:** 10.3390/jcm15031220

**Published:** 2026-02-04

**Authors:** Krzysztof Dobrzeniecki, Zuzanna Poczta, Monika Kacprzak, Anna Kasprzyk, Jadwiga Kolasińska, Małgorzata Mizgier, Magdalena Pisarska-Krawczyk, Katarzyna Plagens-Rotman, Witold Kędzia, Grażyna Jarząbek-Bielecka

**Affiliations:** 1 Department of General and Developmental Sexology, Gynecology Clinic, Poznan University of Medical Sciences, 60-535 Poznan, Polandkpr.pielegniarstwopolskie@interia.eu (K.P.-R.); witold.kedzia@ump.edu.pl (W.K.); grajarz@tlen.pl (G.J.-B.); 2 Department of Sports Dietetics, Faculty of Health Sciences, University School of Physical Education in Poznan, 61-871 Poznan, Poland; mizgier@awf.poznan.pl; 3 Nursing Department, Uniwersytet Kaliski, 62-800 Kalisz, Poland; magmp@op.pl

**Keywords:** human sexual development, psychosexual development, somatic sexual maturation, puberty, precocious puberty, delayed puberty, gender identity, sexual behavior

## Abstract

Human sexual development begins in the prenatal period and continues throughout life, shaped by both biological and psychosocial factors. Somatic development leads to reproductive maturity through several stages regulated by the hypothalamic–pituitary–gonadal axis. Psychosexual development, described in classical theories such as Freud’s and in contemporary models, emphasizes the development of gender identity and sexual behaviors from infancy through early and middle childhood into adolescence, a period characterized by the integration of sexual and emotional components. This developmental trajectory evolves from a biologically driven process into a conscious, socially shaped phenomenon through concretization, mentalization, and socialization. To synthesize current knowledge, this paper is based on a literature review conducted across multiple databases, with studies selected and evaluated for relevance to both somatic and psychosexual development. Understanding the dynamics of these processes is essential for clinical practice, sexual education, and health prevention. It emphasizes integrated clinical practices that employ a multidisciplinary approach, incorporating both medical treatment and psychological support, particularly in the care of children and adolescents with disorders of sexual development. This article presents a comprehensive overview of human sexual development from the prenatal period through adolescence, considering its somatic and psychosexual aspects.

## 1. Introduction

Sexuality is an integral component of human existence, influencing personal identity formation, interpersonal relationships, and societal values and norms [1]. Sexual development refers to the lifelong process through which human sexuality evolves and manifests in diverse forms across different stages of life [2]. It encompasses both biological development, characterized by physical and hormonal changes, and psychosexual development, involving the development of gender identity and gender-role behavior [3].

Biological sexual development comprises physiological and morphological processes leading to sexual maturity and reproductive capacity, highlighting the role of genetic and neuroendocrine factors in determining biological sex. Psychosexual development is a biopsychosocial process integrating biological, psychological, and social influences that shape an individual’s sexual identity, behaviors, and experiences [4]. It includes the formation of attitudes, emotions, needs, and behaviors associated with gender and sexuality.

Although sexual development is often described in separate biological and psychological frameworks, these processes are deeply interconnected. Their origins can be traced to the prenatal period, when the biological foundations of sex differentiation are established, and continue through childhood and adolescence as psychological and social dimensions of sexuality emerge and consolidate [5]. However, much of the existing literature addresses somatic sexual maturation and psychosexual development in isolation or focuses narrowly on specific developmental periods, limiting a comprehensive understanding of sexual development as a continuous, integrated process.

Moreover, current debates on adolescent psychosexual development highlight the growing influence of shifting sociocultural factors. Gender diversity challenges traditional approaches, while widespread digital media shapes how young people explore sexual identity and form intimate relationships by enabling new forms of communication and relationships. Together, these changes, coupled with evolving cultural attitudes toward sexuality, raise new challenges in evaluating how biological, psychological, and social dimensions interact across adolescent development.

Consequently, there remains a need for an integrative synthesis that bridges biological and psychosexual perspectives across successive developmental stages. An approach that considers somatic sexual maturation alongside psychosexual development allows for a more complete understanding of human sexuality, emphasizing the dynamic interplay between biological maturation, psychological development, and social context. Such an integrative perspective is particularly relevant for clinical practice, sexual education, and preventive health care, where fragmented conceptualizations may hinder holistic assessment and intervention [6].

The objective of this narrative review is to provide a comprehensive and integrative overview of human sexual development by examining both somatic and psychosexual dimensions across key developmental stages. By synthesizing biological, psychological, and social perspectives within a unified developmental framework, this article aims to address gaps in the existing literature and to support a more cohesive understanding of healthy sexual development relevant to clinicians, educators, and researchers.

## 2. Methods

This article is based on a review of the literature conducted by the authors to synthesize current knowledge on human sexual development from both somatic and psychosexual perspectives. The literature search was performed independently by three reviewers using the PubMed (Medline), Scopus, Embase, and Web of Science databases. Searches were conducted using combinations of keywords related to sexual development, including: “sexual development”, “psychosexual development”, “puberty”, “biological maturation”, “gender identity”, “sexual differentiation”, “childhood”, “adolescence”, “precocious puberty”, “delayed puberty”, “hypothalamic-pituitary-gonadal axis”, and “sexual behavior”. MeSH keywords and Boolean (AND, OR) operators were employed to enhance the selection of entries.

The literature search and selection process was conducted between September and November 2025. Inclusion criteria comprised peer-reviewed articles published in English or Polish that addressed at least one aspect of human sexual development during childhood or adolescence, with relevance to somatic maturation (e.g., pubertal timing, endocrine regulation, physical development) and/or psychosexual development (e.g., identity formation, sexual behavior, psychosocial correlates). Both empirical studies and theoretical or review articles were considered eligible. The exclusion criteria comprised case reports, conference abstracts, non-peer-reviewed materials, and publications lacking relevance to either somatic sexual maturation or psychosexual development.

Following the initial identification of publications, duplicate records were removed. The remaining articles were screened based on title and abstract to assess their relevance to the objectives of the review. Full-text analysis was subsequently performed for selected publications. Included articles were evaluated with respect to developmental stage addressed, methodological approach, theoretical framework, and relevance to somatic or psychosexual aspects of sexual development.

The final selection of sources was determined through consensus among the reviewers. The analyzed literature was synthesized descriptively, allowing for an integrative presentation of biological, psychological, and social dimensions of sexual development.

## 3. Results

### 3.1. Sexual Development During the Prenatal Period

Human sexual development begins at fertilization with the establishment of chromosomal sex. The presence of sex chromosomes influences gene expression, leading to biological differences between XX and XY embryos even before morphological sex differentiation occurs [7,8]. During the first 7 weeks after fertilization, the embryo is bipotential. Gonadal sex determination then follows.

In XY embryos, expression of the SRY gene on the Y chromosome initiates a cascade of gene activation, leading to differentiation of Sertoli cells and testicular development. In XX embryos, the absence of the SRY gene allows ovarian differentiation pathways to dominate. From approximately the eighth week of gestation, differentiation of internal and external genital organs begins. In XX fetuses, the fallopian tubes, uterus, and upper two-thirds of the vagina develop, followed by formation of the clitoris, labia, and lower vagina around the eleventh week. In XY fetuses, development of the epididymis and ductus deferens occurs, followed by penile and scrotal formation from the eleventh week onward [9].

During prenatal sexual development, disorders of sex development (DSD) may arise from aberrations in the processes of sex determination and differentiation. This includes changes in chromosomes and gonads, as well as the atypical formation of internal and external genitalia. Clinical manifestations of DSD may include ambiguous genitalia in newborns, delayed or atypical puberty, primary amenorrhea, virilization, or difficulty in determining sex. In the psychosexual development of patients with DSD, the level of androgens during the prenatal period is crucial, as it can influence the formation of gender identity and sexual behavior [3].

### 3.2. Physiological Puberty in Girls

Physiological puberty in girls involves coordinated morphological and hormonal changes leading to sexual maturity. Puberty begins with reactivation of the hypothalamic–pituitary–gonadal (HPG) axis, resulting in pulsatile secretion of gonadotropin-releasing hormone (GnRH), followed by increased luteinizing hormone (LH) and follicle-stimulating hormone (FSH) secretion and ovarian sex steroid production (gonadarche). Gonadal hormones stimulate the development of secondary sexual characteristics and exert feedback regulation on the hypothalamus and pituitary [10,11].

The first clinical sign of puberty in girls is breast development (thelarche), typically occurring between 8 and 12 years of age, with variability influenced by genetic, hormonal, and environmental factors [12,13]. Pubarche, the appearance of the first pubic hair in girls, usually follows thelarche and results from adrenal androgen production (adrenarche) during childhood. Adrenarche begins several years before the emergence of pubic hair, typically around the age of 6 [14].

Menarche, the first menstrual period, occurs on average 2–3 years after breast development (thelarche), most commonly between 12 and 13 years of age, though significant individual variability exists. Initial menstrual cycles following menarche are often irregular and characterized by high variability in cycle length and frequent anovulatory cycles. Most cycles in the first year fall within the 21–45 day range, but both shorter and longer intervals between bleeding episodes may occur. Over time, cycles become more regular, resembling those of adult women [15,16]. Monitoring menstrual disorders, including dysmenorrhea, during puberty is important for early diagnosis and intervention [17,18,19].

Anatomical changes occurring in adolescent girls also include the growth and differentiation of the external genitalia, as well as structural changes in the hymen. During puberty, there is an increase in the length and width of the labia minora, an increase in the adipose tissue of the labia majora, and the clitoris becomes more prominent [20]. Internal reproductive organs, such as the uterus and ovaries, also increase in size and maturity, which can be assessed using ultrasonography [21].

The age of puberty onset may vary depending on ethnicity and factors such as obesity or stress [22,23,24]. An American cohort study of over 48,000 girls from various Asian, Hawaiian, and Pacific Islander groups showed that the age of onset of puberty (thelarche and pubarche) varied by more than a year between ethnic subgroups. The earliest puberty was observed in girls of Indian, Hawaiian, and South Asian descent, while the latest was observed in girls of East Asian descent (Chinese, Japanese, Korean). These disparities persisted after adjusting for BMI, suggesting the influence of genetic, environmental, and lifestyle factors [22,25]. Recent studies further confirm that Black and Hispanic girls tend to undergo puberty earlier than White girls, and that both environmental and genetic factors may influence this. In addition, girls who are obese and exposed to psychosocial stress may experience thelarche and menarche earlier, which is associated with the consequences of accelerated puberty [23,24,26,27].

### 3.3. Physiological Puberty in Boys

Puberty in boys is a complex physiological and morphological process involving the attainment of reproductive capability and the development of sexual organs. Similar to puberty in girls, its onset is associated with the reactivation of the HPG axis, which triggers pulsatile secretion of GnRH and a subsequent rise in LH and FSH levels, followed by the onset of androgen production by the testes (gonadarche) [22].

The first signs of puberty in boys typically appear between 9 and 14 years of age [23]. Numerous studies have highlighted that the timing of pubertal onset varies across different ethnic and racial groups and is influenced by a combination of environmental, genetic, and socioeconomic factors. Black individuals tend to enter puberty at younger ages, whereas White and Hispanic individuals generally experience pubertal onset later [28]. Moreover, a recent retrospective cohort study demonstrated variability in pubertal timing across different ethnic groups, including Asian American, Native Hawaiian, and Pacific Islander populations. The study suggested that Native Hawaiian and Pacific Islander boys exhibited the earliest onset of both pubarche and gonadarche among all examined subgroups, whereas Chinese boys showed the latest initiation of these pubertal changes [25].

The marker of pubertal onset in boys is testicular enlargement above 4 mL, resulting from the development of the seminiferous tubules. Subsequently, the scrotal skin becomes darker, thinner, and more wrinkled. Penile growth, including increases in length, circumference, and glans size, follows testicular enlargement [24]. The first conscious ejaculation (spermarche) typically occurs at a mean chronological age of 13.5 years [29]. The development of pubic hair (pubarche) occurs alongside testicular growth and depends on increased secretion of adrenal androgens (adrenarche) [30]. Adrenarche begins around the age of 6, several years before the activation of the HPG axis (gonadarche), and is also responsible for stimulating the sebaceous and sweat glands [31]. Approximately 2 years after pubarche, the growth of facial and chest hair can be observed. The pubertal growth spurt occurs after reaching a testicular volume of 8–10 mL, driven by interactions between sex steroids, growth hormone, and IGF-1. Following this growth spurt, the larynx, cricothyroid cartilage, and laryngeal muscles enlarge, leading to a voice break, which typically occurs around the age of 13.9 years [32].

Pubertal gynecomastia is a benign and transient condition, occurring in approximately half of adolescent boys, that involves enlargement of glandular breast tissue. It is caused by an imbalance between androgen and estrogen levels, and resolves spontaneously in most cases [33].

In addition to somatic maturation, puberty in boys is associated with significant psychological changes, such as development of personal and sexual identity, advanced abstract thinking, and self-reflection, as well as heightened risk-taking behaviours [34,35]. Changes in pubertal timing could affect psychosocial development, as late pubertal onsets have been correlated with increased depressive symptoms [36]. On other hand, studies suggested that early pubertal onsets in boys are linked to higher crime and delinquency [37]. Therefore, both early or late pubertal maturation may increase the risk for psychological distress and further influence psychosocial adjustment.

### 3.4. Methods for Estimating Biological Maturation

Numerous methods have been developed to determine an individual’s stage of biological maturation. The assessment of sexual maturation is based on the development of male and female secondary sexual characteristics, most commonly evaluated using the Marshall and Tanner classification system. The five-stage Tanner classification consists of two sex-specific scales: one assessing genital development in boys and breast development in girls, and the other evaluating pubic hair growth in both sexes [38]. Despite its simplicity and non-invasive nature, the use of the Tanner classification could be limited by interobserver variability and self-assessment bias [39]. Moreover, Tanner staging was originally developed using homogeneous, predominantly White European populations, which limits its generalizability to diverse populations [40]. Anthropometric measures serve as indicators of somatic maturation, allowing researchers to estimate growth patterns and determine the timing of the peak height velocity [41]. Another important indicator of biological maturation is the assessment of skeletal age, which evaluates bone development through X-rays of the left hand and wrist using the Greulich and Pyle atlas. Additionally, dental radiography can be employed as a supportive measure to assess the stage of biological maturation [42]. Radiographic techniques raise ethical questions concerning the use of ionizing radiation on adolescent populations, asserting that it is only justified for clinical diagnosis rather than research. On the other hand, anthropometric methods are less invasive; however, they lack precision due to inherent predictive errors, often underestimating early maturers and overestimating late developers [43].

### 3.5. Precocious Puberty

Precocious puberty is defined as the development of secondary sexual characteristics in girls younger than 8 years old and boys younger than 9 years old [44]. Depending on the underlying pathophysiological mechanism, two main types are distinguished: gonadotropin-releasing hormone-dependent (central) and gonadotropin-releasing hormone-independent (peripheral) [45].

**Scheme 1:**
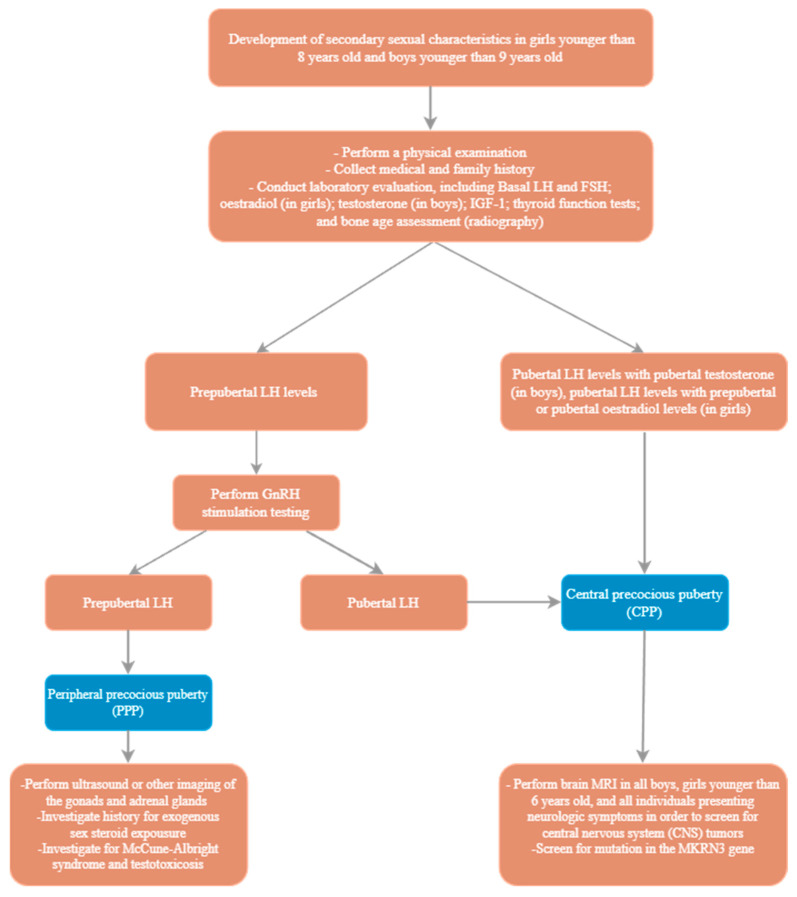
Diagnostic workup methods for precocious puberty.

Central precocious puberty (CPP) results from premature activation of the HPG axis, leading to early development of secondary sexual characteristics. CPP occurs predominantly in girls, in whom it is typically idiopathic. In contrast, there is usually an identifiable underlying pathology in boys, such as hypothalamic hamartomas, gliomas, head trauma, or genetic disorders [46,47]. Peripheral precocious puberty (PPP) arises from the production of sex steroids independent of the HPG axis activation. These steroids are generally produced by the gonads or adrenal glands or result from exogenous exposure. PPP may present as isosexual (consistent with the individual’s biological sex) or heterosexual (inconsistent with biological sex, characterized by feminization in boys or virilization in girls) [48]. Distinctive causes of PPP include McCune–Albright syndrome, testotoxicosis, and recurrent ovarian cysts. McCune–Albright syndrome is characterized by a triad of symptoms: precocious puberty, fibrous dysplasia of the bones, and café-au-lait skin spots, with the possible coexistence of other endocrinopathies [49]. In turn, testotoxicosis in boys causes androgenization as early as around the age of two, with increased levels of sex steroids and low gonadotropins [50]. Scheme 1 summarizes diagnostic workup methods for precocious puberty.

Precocious puberty leads to accelerated skeletal maturation and premature epiphyseal closure, resulting in tall stature during childhood but short stature in adulthood [45]. Emotional development usually remains consistent with chronological age, which could result in potential psychosocial challenges.

### 3.6. Delayed Puberty

Delayed puberty is defined by the absence of breast development by 13 years of age in girls and the absence of testicular enlargement (testicular volume < 4 mL) by 14 years of age in boys. These criteria correspond to an age exceeding 2 standard deviations above the average age of puberty onset in the general population. Clinically, the development of pubic hair is not considered, as it can be a result of adrenarche, independent of the activation of the HPG axis [51].

The most common cause of delayed puberty is constitutional delay of growth and puberty (CDGP), which primarily results from genetic predisposition and often exhibits a familial pattern [52,53]. Despite being the most common cause of delayed sexual maturation in both sexes, CDGP can only be established after alternative pathological causes have been excluded. The differential diagnosis of CDGP can be broadly categorized into three main groups: hypergonadotropic hypogonadism, permanent hypogonadotropic hypogonadism, and transient hypogonadotropic hypogonadism [51].

**Scheme 2:**
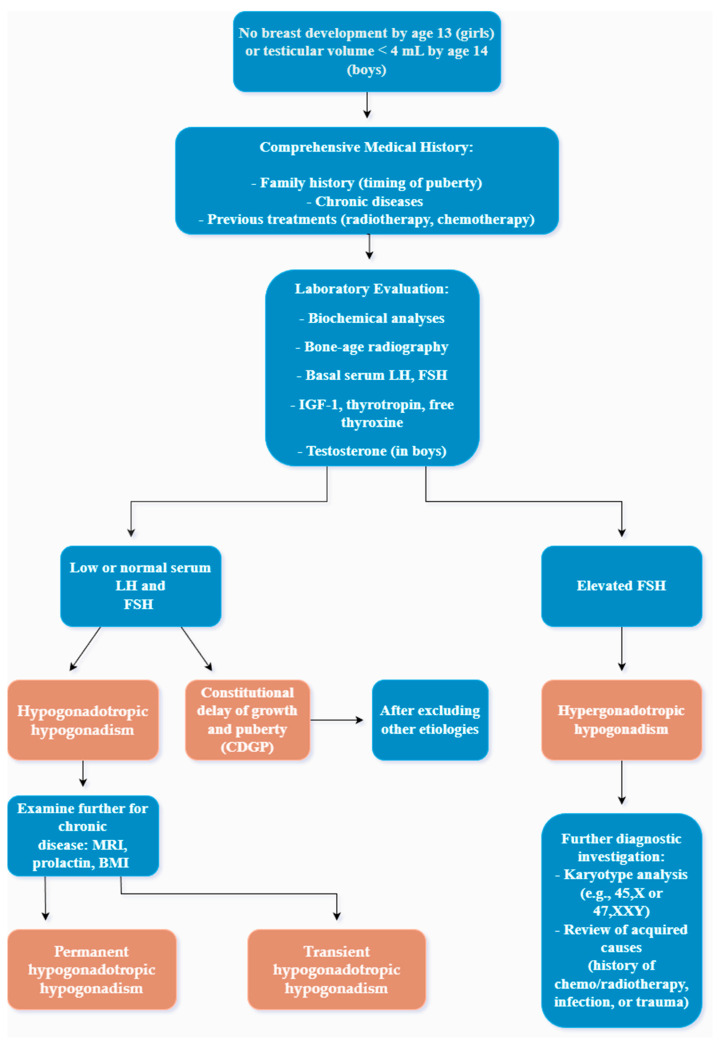
Diagnostic workup methods for delayed puberty.

Hypergonadotropic hypogonadism is characterized by elevated levels of LH and FSH due to a lack of negative feedback from the gonads. It can be congenital, resulting from gonadal dysgenesis (e.g., Turner syndrome in girls or Klinefelter syndrome in boys). It can also be acquired, stemming from gonadal damage, such as from radiation or chemotherapy. Permanent hypogonadotropic hypogonadism is characterized by low levels of LH and FSH due to disorders of the hypothalamus or pituitary gland and may be caused by tumors or infiltrative diseases of the central nervous system, GnRH deficiency (isolated hypogonadotropic hypogonadism, Kallmann’s syndrome), combined pituitary hormone deficiency, or chemotherapy or radiation therapy. Transient hypogonadotropic hypogonadism (functional gonadotropin deficiency), where the delay in sexual maturation is caused by a delayed maturation of the HPG axis secondary to systemic illness (inflammatory bowel disease, celiac disease, anorexia nervosa or bulimia), hypothyroidism, or excessive physical exercise [51,54]. Diagnostic workup methods for delayed puberty are presented in Scheme 2.

Delayed puberty can have significant implications for the patient’s psychological profile and mental health. It is associated with a heightened risk of diminished self-esteem, social isolation, and elevated levels of psychosocial stress. Both sexes may experience dissatisfaction with their appearance, particularly in terms of height and the development of secondary sexual characteristics, which can lead to withdrawal from peer interactions and difficulties in psychosocial functioning. Consequently, it is imperative that patients receive a timely diagnosis, tailored clinical management, and comprehensive psychological support [55,56].

### 3.7. Psychosexual Development

For many years, it was assumed that sexual behaviors, needs, and capacities emerge only with the onset of physiological sexual maturation. However, Sigmund Freud’s psychoanalytic theory of psychosexual development fundamentally challenged this view. Freud posited that humans are born with an inherent sexual drive, which, over the course of development, becomes increasingly focused on specific erogenous zones, including the mouth, anus, and genitals. This perspective disputed the traditional notion of childhood as an asexual stage.

Contemporary understanding recognizes that human sexuality begins at conception and evolves throughout the lifespan, shaped by an interplay of biological, environmental, and sociocultural factors. The two-component ecological model conceptualizes sexuality as an inherent human attribute—a universal yet dynamic drive influenced by both innate and acquired factors. Innate factors include genetic determinants that shape anatomical structures and physiological functions, enabling the experience of pleasure through stimulation of erogenous zones and the capacity for orgasm. Acquired factors encompass individual experiences across the lifespan, including sexual and relational experiences. Pleasant experiences, made possible by innate capacities, become associated with specific behaviors, thereby reinforcing sexual motivation. Within this framework, sexuality is understood as both constant—present throughout life—and dynamic, evolving through continuous interaction between innate and acquired influences, with the relative contribution of each varying across developmental stages [57,58].

According to a theoretical framework proposed by Martin Seligman, psychosexual development progresses through a series of interconnected hierarchical layers, each building upon the preceding one. These layers encompass gender identity, sexual orientation and preferences, the expression of gender roles, and sexual behaviors [2,3].

John Bancroft’s eclectic and interactional model of sexual development also represents an attempt to integrate biological, psychological, and socio-cultural factors within a theory of human sexual development. The model proposes the existence of three main developmental strands of sexuality: (1) sexual differentiation toward male or female and the development of gender identity; (2) sexual responsiveness; and (3) the capacity to form close dyadic relationships. Throughout most of childhood, these strands develop relatively independently of one another. Toward the end of childhood and during early adolescence, however, they begin to interconnect and mutually influence each other, which ultimately leads to the formation of a sexually integrated adult individual [58].

#### 3.7.1. Psychosexual Development in Infancy and Early Childhood

According to theories of sexual development, early childhood (usually described as ages 0–3) constitutes a crucial foundation for later psychosexual development. During this time, the core of gender identity is formed and, according to some theorists, sexual orientation may also begin to develop. Around the age of three, a child develops the ability to identify and name their own gender, recognize factors determining gender membership, and apply basic gender stereotypes [59].

The period of early childhood—corresponding to the preoedipal stage in Freud’s classification—is characterized by a strong emotional and physical bond between the child and the mother. According to Otto Kernberg’s object relations theory, sensory experiences arising from contact with the mother (such as breastfeeding or caregiving) lay the foundation for later capacities to experience closeness, trust, and pleasure in relationships with others, including sexual ones. The mother functions as the first object that evokes sensory stimulation and emotional arousal in the child, while the influence of others (peers, extended family, institutions) is less significant at this stage. Empirical findings support the existence of early sexual and sensual responsiveness in infancy, showing that the capacity for sexual response is present from birth [2]. In the following months, children display sexual behaviors in the form of spontaneous body exploration. Infants aged 6 to 11 months touch their genitals, and by the end of the first year, their movements become increasingly purposeful and direct [52,60]. Between 2 and a half and 3 years of age, children begin to show rhythmic self-stimulatory movements that resemble adult-like patterns of genital stimulation [2].

From the modern perspective of John Bancroft’s contemporary eclectic model, at this stage of development the presence of early sexual and sensual responsiveness is not yet interpreted by the child as sexual, nor is it associated with erotic meaning or with a relationship with another person. This is consistent with the assumptions of the model, according to which sexual responsiveness may develop independently of the attribution of sexual meaning and of the capacity to form dyadic relationships. Although children observe relationships between men and women relatively early, at this stage they do not yet attribute a sexual character to such relationships. For this reason, the main strands of sexual development remain independent [58].

#### 3.7.2. Psychosexual Development in Middle Childhood

During middle childhood (ages 4–6), children’s interest in sexuality and sexual activity increases significantly [2]. This stage marks a period in which children begin to engage with topics directly related to sex, love, and the human body. These emerging interests are supported by cognitive, emotional, and social development, as children become more curious, socially aware, and capable of forming relationships with others [61]. At this age, children also develop a sense of gender constancy, understanding that a person remains the same gender despite changes in their appearance. From Bancroft’s biopsychosocial perspective, the increase in sexual curiosity reflects the further development of the strand of sexual responsiveness; however, these activities are not yet integrated with sexual meaning or with gendered interactions between peers [58].

According to the Freudian psychoanalytic theory, this stage corresponds to the Oedipal period, during which a child’s sexuality is shaped not only by the mother but also by the entire family system and relationships with peers. During the Oedipal stage, a child’s mental activity focuses on conflicts and tensions related to relationships with parents of both the same and opposite sex. It is at this time that the first signs of the Oedipus complex appear: the child identifies with the parent of the same sex while competing for the attention and affection of the parent of the opposite sex, toward whom they feel sexual desire. This conflict is crucial for the development of the child’s capacity to form mature relationships, as they begin to understand that their parents (as well as adults in general) are sexually unavailable, and that future partners should be sought outside the family, among peers of a similar age [62]. Sexual arousal initially directed toward parents gradually shifts into peer-oriented activity.

Children engage in four types of sexual behaviors: (a) masturbatory, which include all types of self-stimulation; (b) exploratory—actions aimed at discovering anatomical differences between males and females, including early forms of exhibitionism and voyeurism (exposing one’s own genitals or observing other naked bodies); (c) interactive, typically in the form of childhood sexual play, in which children may role-play adult sexual or domestic scenarios; and (d) creative, which include expressing sexual interest through drawings, stories, or rhymes of erotic content. Observational studies indicate that during the preschool years, the most frequently observed sexual behaviors include touching one’s own genitals, touching the mother’s or other women’s breasts, and looking at nude individuals [62,63].

Although such behaviors are characteristic of this developmental stage, they occur within a framework shaped by social and cultural norms. It is important to recognize that sexual behaviors, including masturbation, may occasionally present in pathological forms, associated with psycho-emotional disturbances, adverse family circumstances, or, in some cases, as indicators of sexual abuse [64]. Additionally, in certain individuals, early sexual behaviors may be linked to obsessive-compulsive tendencies or persistent sexual preoccupations later in development [65]. Parents, caregivers, and educators play a critical role in guiding children’s understanding of privacy, consent, and appropriate sexual conduct, thereby helping them distinguish between normative exploratory behaviors and actions that may be potentially harmful to themselves or others. As children progress through middle childhood, they gradually acquire the cognitive and social capacities necessary to comprehend societal sexual norms and regulate their behavior accordingly [61].

#### 3.7.3. Psychosexual Development in Late Childhood

Late childhood (approximately ages 7–12) is referred to in psychoanalytic theories as the latency phase, due to a temporary redirection of energy away from sexuality toward new developmental tasks, such as school performance and peer cooperation. Freud described this period as one of relative dormancy in sexual drive, resulting from the repression of early impulses and the absence of a specific, dominant erogenous zone. Sexual energy is redirected toward other activities, such as learning, sports, or hobbies, which allows for the attenuation of Oedipal desires and feelings toward the parents, the development of a more stable and supportive relationship with them, greater involvement in peer relationship and the emergence of new interests. Children in this stage typically form same-sex peer groups and show limited romantic or sexual interest. Sexuality, however, remains active at an unconscious level, forming the foundation for later development [66]. At this age, there is often a disintegration of sexuality—an observable separation between sexuality and aggression versus tenderness and affection. Among boys, the elements of sexuality and aggression are more distinct, often expressed through sexual jokes or vulgar language, which serve as a safe outlet for sexual tension. In contrast, girls display a predominance of tenderness and a desire for close emotional relationships, while simultaneously avoiding or denying aspects related to sexual desire or behavior [62].

Contemporary research challenges the idea of latency as a period of sexual inactivity, emphasizing that biological, cognitive, and social changes during these years also shape gender identity and understanding of social and sexual norms. During late childhood, children’s sexual knowledge expands alongside cognitive maturation, enabling greater understanding of reproductive processes, gender identity, and interpersonal relationships. Age-appropriate sexual behaviors—including body curiosity, self-exploration, interest in reproduction and sexuality, and masturbation—are considered normative aspects of healthy psychosexual development [67].

Late childhood is marked by substantial maturation of executive functions, particularly inhibitory control, working memory, and sustained attention, supporting improved self-regulation, academic functioning, and social competence. Increasing behavioral control contributes to the reduced public expression of sexual behaviors, as children internalize social norms and increasingly regulate sexual curiosity through privacy. Emotional regulation also advances during this period, though it remains influenced by parenting practices and peer experiences. Growing capacity to recognize and modulate emotions supports more complex social interactions and shapes how sexual curiosity and relational boundaries are managed [68].

Peer relationships represent a central developmental context in late childhood. The period is marked by pronounced gender segregation, followed by a gradual transition toward mixed-gender interactions approaching adolescence. Socialization pressures from families, schools, peers, and media reinforce gender norms, which are increasingly internalized by approximately 10 years of age. Friendship quality and stability increase across late childhood, with relationships becoming more reciprocal, intimate, and enduring [69].

From the perspective of Bancroft’s eclectic model, a better understanding of the significance of sexual behavior and gender roles is associated with the development of the strand of sexual responsiveness and sexual identity, while the strand of dyadic relationships is developed in peer relationships. However, these strands remain relatively separate until adolescence [58].

#### 3.7.4. Psychosexual Development in Adolescence

Adolescence is a critical period characterized by sexual maturation, identity formation, and the beginning of adult sexual behavior. It involves biological, psychological, and social changes influenced by puberty, hormone changes, cognitive development, and evolving social relationships.

In Freud’s theory, adolescence marks an intensification of psychosexual development leading to mature, genital sexuality. The key tasks include integrating sexual and emotional components, resolving parental conflicts, and establishing identity within the peer group. This period is marked by the reactivation of previously latent sexual drives, which become predominantly directed toward peers rather than toward internalized or autoerotic fantasies. Instead of focusing on pleasure derived from one’s own body, sexual interest becomes oriented toward behaviors involving a partner. Successful resolution results in a coherent sexual identity and the capacity for intimacy and love [70].

During adolescence, tensions with parents and the search for autonomy facilitate a gradual shift toward peer groups, which serve as arenas for developing sexual attitudes and behaviors [71]. Early adolescence is typically characterized by same-sex peer groups that function as safe spaces for exploring closeness and identity. Relationships formed during this period tend to be unstable and strongly oriented toward either sexual desire or tenderness. Later adolescence introduces mixed-gender groups and more stable romantic relationships.

As in previous developmental phases, the course of psychosexual development differs between boys and girls. Among boys, rapid hormonal and physical changes, not followed by corresponding emotional development and the ability to control one’s behavior, lead to strong sexual tension and immature, uncontrolled sexual expressions such as masturbation, voyeurism, or consumption of erotic material. In the second half of adolescence, growing awareness of bodily changes leads to a desire to release tension through contact with another person, though this is not necessarily accompanied by emotional intimacy or the capacity for romantic attachment. Among girls, sexual needs during early adolescence are often unconscious, accompanied instead by a strong drive for emotional closeness. Sexual disintegration in this group may manifest as a denial of one’s own sexuality, sometimes described as the asceticism of adolescence. In late adolescence, sexual arousal becomes more conscious and accepted, leading to greater interest in sexual relationships. In both genders, the separation of sexual and emotional components gradually diminishes with maturity, culminating in full integration and the capacity to realize both aspects within a single, intimate adult relationship. Sexual behaviors during adolescence include masturbation, which predominates and becomes more frequent in early adolescence and is gradually replaced by partnered activities, such as petting or genital sexual activity [72,73,74].

From a contemporary biopsychosocial perspective, adolescent psychosexual development is understood as the product of dynamic interactions among biological maturation, cognitive development, and social context. Pubertal hormonal changes, including increases in gonadal steroids, contribute to heightened sexual drive and interest, while ongoing maturation of prefrontal cortical networks supports the gradual development of impulse control, planning, and emotional regulation. The temporal imbalance between subcortical reward systems and cognitive control mechanisms can explain the intensity, variability, and sometimes risk-prone nature of adolescent sexual behavior [58,75].

Adolescence is a critical period for the formation of sexual identity, orientation and preferences, as adolescents experience increased awareness of their sexual selves and begin to explore romantic and sexual relationships. This process unfolds along diverse trajectories and is influenced by individual temperament, social support, and sociocultural acceptance.

Environmental influences play a central role in shaping sexual attitudes, behaviors, and identity formation. Family communication patterns, peer norms, romantic experiences, cultural expectations, and media exposure, including digital and social media, significantly affect how adolescents interpret and express emerging sexuality. Peer groups and romantic partners serve as key contexts for learning relational skills, negotiating consent and boundaries, and integrating sexual desire with emotional intimacy [75].

In Bancroft’s model, adolescence is considered a time of gradual convergence of three strands of sexuality. Sexual identity, sexual behavior, and dyadic relationships become increasingly consistent with each other, building an image of integrated sexuality, where sexual behavior is carried out in mature relationships, remaining consistent with sexual identity [58].

An overview of the key milestones in somatic and psychosexual development from early childhood through adolescence is presented in Table 1.

### 3.8. The Transformation of Sexual Need and the Formation of Mature Sexuality

The sexual need, understood as a fundamental drive to function in relationships that allow for both sexual arousal and release, as well as emotional bonding, evolves from a biological drive into a conscious, socially shaped phenomenon. This transformation occurs through three interrelated processes: concretization, mentalization, and socialization. Concretization denotes the developmental shift from less efficient to more mature means of satisfying sexual needs, leading to higher levels of satisfaction. In the process of learning, more gratifying behaviors are reinforced, while less gratifying behaviors gradually diminish. An example is the developmental progression from autoerotic behaviors to dyadic sexual activity. Mentalization is the process of becoming aware of one’s sexual needs and understanding one’s experiences in sexual terms. It denotes the ability to reflect upon one’s sexuality, comprehend underlying motivations, desires, and emotions, and integrate them into one’s self-concept. Socialization refers to the shaping of sexual drives through social norms, cultural values, and behavior patterns accepted within a given society. It serves as a mechanism of adaptation to culture and society through the internalization of norms concerning sexuality. During sexual socialization, individuals learn culturally appropriate sexual behaviors, acquire gender roles, and come to understand the boundaries of what is considered permissible, desirable, or unacceptable within their culture. These processes jointly determine individual patterns of sexual functioning and account for diversity in sexual preferences and behaviors [76,77,78].

## 4. Conclusions

Human sexual development includes both somatic aspects, related to physiological and hormonal maturation, and psychosexual aspects, which shape sexual orientation, gender identity, and sexual behaviors. This process unfolds gradually from the prenatal period through adolescence. Its healthy progression relies on interaction of biological, emotional, and social components, highlighting the importance of an interdisciplinary approach to human sexual development. Integrating perspectives from medicine, psychology, and social sciences allows for effective monitoring, early intervention, and support for healthy psychosexual and somatic growth throughout childhood and adolescence. This integrative perspective underscores the necessity of interdisciplinary approaches that combine medical, psychological, and social-scientific expertise. Such approaches are particularly critical in clinical contexts, including the care of individuals with disorders of sex development or atypical pubertal timing, where somatic variations may have profound psychosexual and psychosocial consequences. Early, coordinated interventions can support identity formation, emotional well-being, self-esteem, and social functioning, thereby improving long-term outcomes. Future research may include longitudinal studies that examine the bidirectional relationships between biological maturation and psychosexual development across developmental stages. Special attention to vulnerable populations, such as those with disorders of sex development or pubertal disorders, will further enhance understanding and inform evidence-based clinical practice, sexual education, and preventive health strategies.

## Figures and Tables

**Table 1:** Key important milestones in somatic and psychosexual development from a clinical perspective.

Developmental Stage	Psychosexual Development Milestones	Somatic Sexual Maturation Milestones
Early childhood (0–3 years)	Formation of the core of gender identity; understanding basic gender stereotypes [59]. Spontaneous body exploration; masturbatory-like behaviors [2,52,60]. A child’s mother is the primary source of sensory and emotional stimulation; others have less influence [61]. Gender identity, sexual responsiveness and forming sexual relations remain not yet integrated [58].	
Middle childhood (4–6 years)	Development of gender stability and constancy [61]. Significant increase in interest in sexuality and sexual activity [2,61]. Engaging in sexual behaviors: masturbatory, exploratory, interactive and creative [62,63]. Gradual ability to comprehend social norms related to sexual behaviors [61]. Sexual arousal begins shifting from parents to peers (early peer-directed sexual curiosity).	Increased secretion of adrenal androgens (adrenarche) [30].
Late childhood (7–12 years)	Refinement of gender roles understanding [67]. Internalization of social norms regarding sexual behavior [68]. Engaging in sexual activities in private rather than publicly [68]. Increased importance of peer relationships [69]. Forming of mix-gendered groups.	Breast development (thelarche), Tanner Stage B2 [12,38]. Pubic hair growth (pubarche), Tanner Stage P2 [12,38]. Testicular enlargement ≥ 4 mL, Tanner Stage G2 [38].
Adolescence (13–18 years)	Development of sexual identity, orientation and preferences [75]. Increased sexual drive and interest [75]. Shift from masturbatory-like behaviors to dyadic sexual behaviors [55,56,57]. Shift from same-gendered groups to mixed-gendered groups and to more stable relationships [54]. Progressive ability to combine intimacy and sexuality in a single relationship [54]. Integration of three sexuality strands: sexual identity, sexual responsiveness and dyadic relationship [58].	First menstruation (menarche) [12]. The first conscious ejaculation (spermarche) [29].

## Data Availability

No new data were created.

## Abbreviations

The following abbreviations are used in this manuscript:

CDGP	Constitutional delay of growth and puberty
CPP	Central precocious puberty
DSD	Disorders of sex development
FSH	Follicle-stimulating hormone
GnRH	Gonadotropin-releasing hormone
HPG	Hypothalamic–pituitary–gonadal
LH	Luteinizing hormone
PPP	Peripheral precocious puberty
